# Clinical outcomes of botulinum toxin A management for neurogenic detrusor overactivity: meta-analysis

**DOI:** 10.1080/0886022X.2019.1655448

**Published:** 2019-10-10

**Authors:** Shang-Jun Wu, Yu-Qiong Xu, Zheng-Yan Gao, Zhi-Peng Wang, Feng Zhao, Lin Liu, Sheng Wang

**Affiliations:** a Department of Urology, Huangshi Central Hospital, Affiliated Hospital of Hubei Polytechnic University, Huangshi, China;; b Peking University Shenzhen Hospital, Shenzhen, China;; c Department of Urology, The Sixth People's Hospital of Yancheng City, Yancheng, China;; d Department of Urinary Surgery, Linzi District People’s Hospital, Binzhou Medical University, Yantai, China;; e Department of Transfusion Medicine, Linzi District People’s Hospital, Binzhou Medical University, Yantai, China;; f Department of Biotherapy, Linzi District People’s Hospital, Binzhou Medical University, Yantai, China;; g Medical Intensive Care Unit, PKUCare Luzhong Hospital, Zibo, China

**Keywords:** Neurogenic detrusor overactivity, botulinum toxin A, urinary incontinence, adverse effects, meta-analysis

## Abstract

The aim of this work was to evaluate the efficacy and safety of botulinum toxin A (BTX-A) treatment in patients with neurogenic detrusor overactivity. PUBMED, EMBASE, and Cochrane Library were identified on 13 May 2017 to identify relevant randomized controlled trials. All data obtained were analyzed using Stata 12.0. Five randomized controlled trials were included in this study. Compared to placebo, the BTX-A groups had significantly fewer urinary incontinence (UI) episodes per day and per week (BTX-A with 300 U for frequency of UI per day at week 2, mean difference (MD): −1.13, 95% confidence interval (CI): −1.89 to −0.37; 200 U; BTX-A with 300 U for frequency of UI per week at week 6, MD: −11.42, 95% CI: −13.91 to −8.93; BTX-A with 200 U for frequency of UI per week at week 6, MD: −10.72, 95% CI: −13.40 to −8.04), increased in maximum cystometric capacity at week 6 (BTX-A with 300 U, MD: 154.88, 95% CI: 133.92–175.84; BTX-A with 200 U, MD: 141.30, 95% CI: 121.28–161.33), decreased maximum detrusor pressure at week 6 (BTX-A with 300 U, MD: −31.72, 95% CI: −37.69 to −25.75; BTX-A with 200 U, MD: −33.47, 95% CI: −39.20 to −27.73). For adverse effects, BTX-A was often associated with more complications and urinary tract infections (BTX-A with 300 U: relative risk (RR):1.42, 95% CI: 1.15–1.76; BTX-A with 200 U: RR: 1.42, 95% CI: 1.11–1.82). This meta-analysis suggests that treatment with BTX-A is effective and safe for neurogenic detrusor overactivity, and recommends using BTX-A with 300 U or with 200 U, as suitable dosage.

## Introduction

Overactive bladder (OAB), defined by the International Continence Society as urgency with or without urinary incontinence (UI), usually associated with frequency and nocturia [1], is a multifactorial and common health disorder associated with detrimental effects on the patients’ quality of life and a huge economic burden [2,3]. OAB includes detrusor instability and hyperreflexia, both of which are described as neurogenic detrusor overactivity (NDO) in neurogenic bladder [4]. NDO is a subtype of OAB due to spinal cord injury (SCI) or multiple sclerosis (MS) [1]. It is characterized by a combination of urinary frequency, urgency, and UI [1]. The urodynamic parameters of patients with NDO include a high transient bladder pressure, low bladder capacity, and increasing UI episodes [5]. Oral anticholinergic drug treatment, frequently combined with clean intermittent catheterization, is considered first-line therapy for UI in these patients [6]. However, many patients discontinue this treatment owing to its inadequate efficacy and/or intolerable adverse events [7,8].

Onabotulinum toxin A, a specific formulation of botulinum toxin A (BTX-A), is a neuromodulator that inhibits vesicle-mediated neurotransmission and reduces muscle spasticity. It has emerged as an effective second-line therapy in the management of NDO, with a recent European consensus giving it a Grade A recommendation for use in this condition [9]. The efficacy of intra-detrusor BTX-A injection in treating NDO was first reported in 2000 [10], when it was shown to significantly decrease UI episodes and improve urodynamic parameters at doses of 200 and 300 U in several randomized placebo-controlled trials [11–14]. However, modulation of neuromuscular transmission may also result in urinary retention, and therefore, the use of BTX-A is still under debate [15].

The goal of this study was to perform a meta-analysis to assess the efficacy and safety of onabotulinum toxin A in treating NDO, in an attempt to resolve some of the current controversies over the use of this drug.

## Materials and methods

### Literature search

The PUBMED, EMBASE, and Cochrane Library databases were browsed until 13 May 2017 to obtain relevant studies that evaluated the efficacy and safety of BTX-A for treatment of NDO. The following keywords, including ‘Botulinum toxin’, ‘Onabotulinumtoxina’, ‘Overactive bladder’, ‘Overactive urinary bladder’, ‘Overactive detrusor function’, ‘Neurogenic detrusor overactivity’, ‘Urinary incontinence’, and ‘Randomized controlled trials’, were used in the above three databases.

### Inclusion and exclusion criteria

The inclusion criteria for this study are (1) UI patients due to NDO, (2) all patients were adults (>18 years), (3) the experimental group was BTX-A, and control group was placebo or other dose of BTX-A, and (4) all studies were randomized controlled trials (RCTs) in English.

The excluded criteria for this study are (1) population with other types of UI, (2) missing value of standard deviations were not acquired, and (3) language is not English.

### Quality assessment

Two reviewers assessed the methodological quality of the included studies using the following criteria: assessment of random sequence generation, allocation concealment, blinding of participants and personnel, blinding of outcome assessment, incomplete outcome data, selective reporting, and other biases. In the Cochrane Collaboration Reviewers’ Handbook for Systemic Reviews of Interventions [16], for each item based on the question, the judgment (‘low risk’ of bias, ‘unclear risk’ of bias, or ‘high risk’ of bias) is followed by a text box, which provides a basis for the description of the design, implementation, or measurement as a judgment.

### Data extraction

This study collected the following information: (1) the characteristics of included studies; (2) research design of included studies based on the Cochrane handbook; (3) we have a predetermined outcomes: frequency of UI per day and per week, maximum cystometric capacity (MCC), maximum detrusor pressure (MDP), and urinary tractinfections (UTIs). All disputes are settled by the corresponding author.

### Statistical analysis

Differences are expressed as relative risk (RR) with 95% confidence interval (CI) [16,17] for dichotomous outcomes and mean difference (MD) with 95% CI [16] for continuous outcomes. Heterogeneity across studies was tested using the *I*^2^ statistic at a significance level of *p* < 0.1. Studies with an *I*^2^ = 0 were considered to have no heterogeneity, while larger *I*^2^ values indicated greater heterogeneity. Studies with an *I*^2^ statistic >50% or *p* < 0.1 were considered to have significant heterogeneity. A fixed-effects model was used if there was no significant heterogeneity. Otherwise, random-effects model were employed in the meta-analysis. Publication bias was qualitative evaluated using funnel plot [16]. Additionally, Egger’s test was employed for quantitative detection bias [16]. All the statistical analyses were performed using Stata 12.0.

## Results

### Study selection and characteristics of individual studies

**Figure 1.**
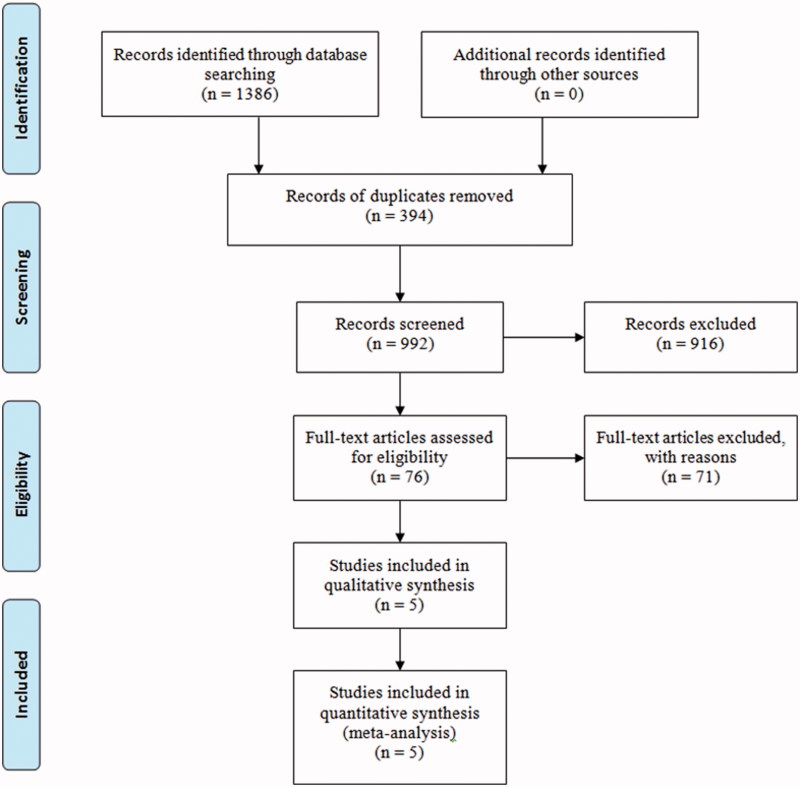
A flow diagram of the study selection process.

The search resulted in 1386 articles. After initial evaluation, 394 studies were removed for being duplicates, 916 for being irrelevant (as determined by reading the abstracts), and 76 studies were excluded for reasons determined by reading the full text. Eventually, five [11–14,18] RCTs were involved in this study (Figure 1). Table 1 showed the basic characteristics of included studies and Table 2 presented the results of quality assessment.

**Table 1.** Characteristics of individual study.

First author	Year	Region	No. of patients (female)	Age, mean (SD)	Design	Classification of urinary incontinence	Basic diseases	Intervention	Follow-up (weeks)
Schurch [11]	2005	Switzerland	59(23)	41	Randomized, doubled-blind	NDO	MS:6, SCI:53	Group1:BTX-A 300U (19); Group2:BTX-A 200U (19); Group3:placebo(21)	6
Cruz [12]	2011	Portugal	275(155)	46(13.1), 44.4(13.9), 46.9(13.4)	Randomized, doubled-blind	NDO	MS:154, SCI:121	Group1:BTX-A 200U(92); Group2:BTX-A 300U(91); Group3:placebo(92)	2,6,12
Herschorn [13]	2011	Canada	57(23)	42.8	Randomized, doubled-blind	NDO	SCI:38, MS:19	Group1:BTX-A 300U(28); Group2:placebo(29)	NA
Ginsberg [14]	2012	USA	416(245)	NA	Randomized, doubled-blind	NDO	MS:227, SCI:189	Group1:BTX-A200U(135); Group2:BTX-A300U(127); Group3:placebo(145)	6
Rovner [18]	2013	USA	691(400)	45.9,45.6,46.2	Randomized, doubled-blind	NDO	MS:103, SCI:138	Group1:BTX-A 200U(227); Group2:BTX-A 300U(223); Group3:placebo(241)	6

NDO: neurogenic detrusor overactivity; MS: multiple sclerosis; SCI: spinal cord injury; BTX-A: botulinum toxin A; NA: not available.

**Table 2.** Quality assessment of individual study.

Author	Year	Random sequence generation	Allocation concealment	Blinding of participants and personnel	Blinding of outcome assessment	Incomplete outcome data	Selective reporting	Other
Schurch [11]	2005	Low	Low	Low	Low	Low	Low	Unclear
Cruz [12]	2011	Low	Low	Low	Low	Low	Low	Unclear
Herschorn [13]	2011	Low	Low	Low	Unclear	Low	Low	Unclear
Ginsberg [14]	2012	Low	Low	Low	Low	Low	Low	Unclear
Rovner [18]	2013	Low	Low	Low	Low	Low	Low	Unclear

### Frequency of UI per day at week 2

**Figure 2.**
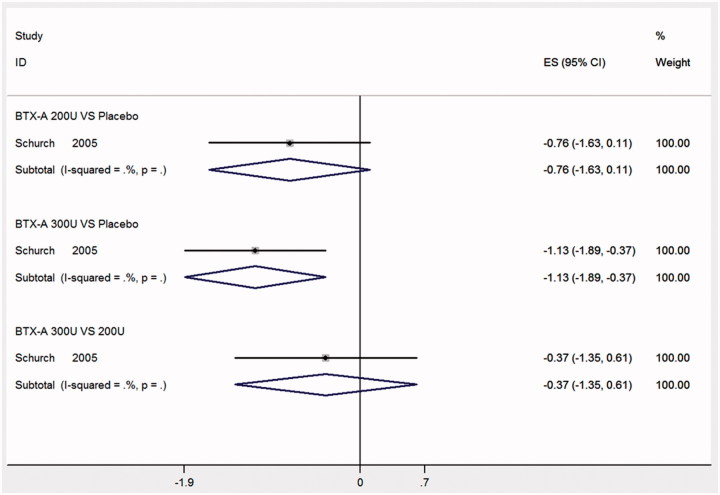
Forest plot of the changes of urinary incontinence episodes per day at week 2.

Only 1 RCT [11] was reported to evaluate frequency of UI per day at week 2. Compared with placebo, BTX-A with 300 U significantly reduced the frequency of UI per day (MD: −1.13, 95% CI: −1.89 to −0.37), but the result of BTX-A with 200 U vs. placebo in the frequency of UI per day (MD: −0.76, 95% CI: −1.63 to 0.11) was not statistical difference in Figure 2. However, the results of BTX-A with 300 U and BTX-A with 200 U (MD: −0.37, 95% CI: −1.35 to 0.61) was not statistical difference.

### Frequency of UI per week at week 6

**Figure 3.**
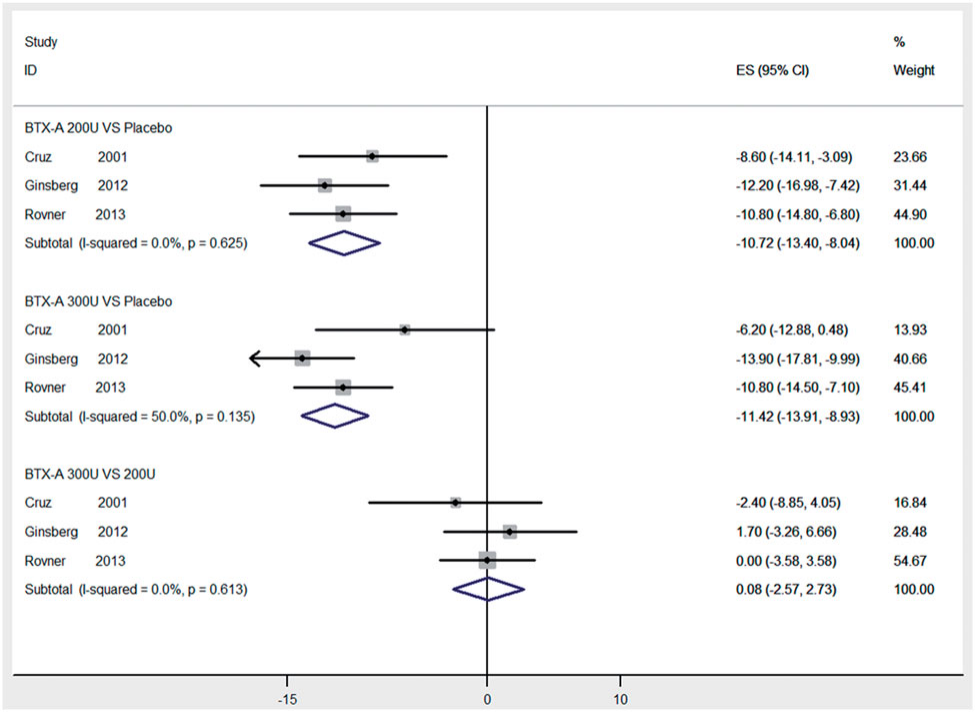
Forest plot of the changes of urinary incontinence episodes per week at week 6.

Three RCTs [12,14,18] were involved in evaluating the frequency of UI per week at week 6. As shown in Figure 3, there were significant reductions in frequency of UI per week (BTX-A with 300 U vs. placebo, MD: −11.42, 95% CI: −13.91 to −8.93; BTX-A with 200 U vs. placebo, MD: −10.72, 95% CI: −13.40 to −8.04, respectively). However, the difference between 300 and 200 U at week 6 was not statistically significant (MD: 0.08, 95% CI: −2.57 to 2.73).

### Maximum cystometric capacity at week 6

**Figure 4.**
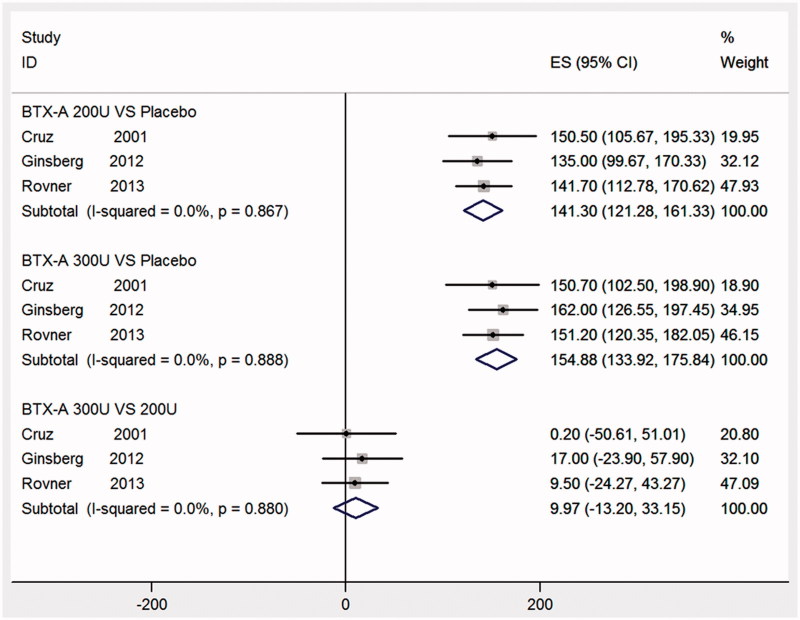
Forest plot of the changes of maximum cystometric capacity at week 6.

Three RCTs [12,14,18], reporting data for MCC at week 6, and were collected in our study. Figure 4 showed that both BTX-A with different dose had statistically significant improvement in MCC at week 6, when comparing BTX-A with 300 U and BTX-A with 200 U with placebo (MD: 154.88, 95% CI: 133.92–175.84; MD: 141.30, 95% CI: 121.28–161.33, respectively). Significant improvements were not seen in a comparison of BTX-A with 300 U and BTX-A with 200 U (MD: 9.97, 95% CI: −13.20 to 33.15).

### Maximum detrusor pressure at week 6

**Figure 5.**
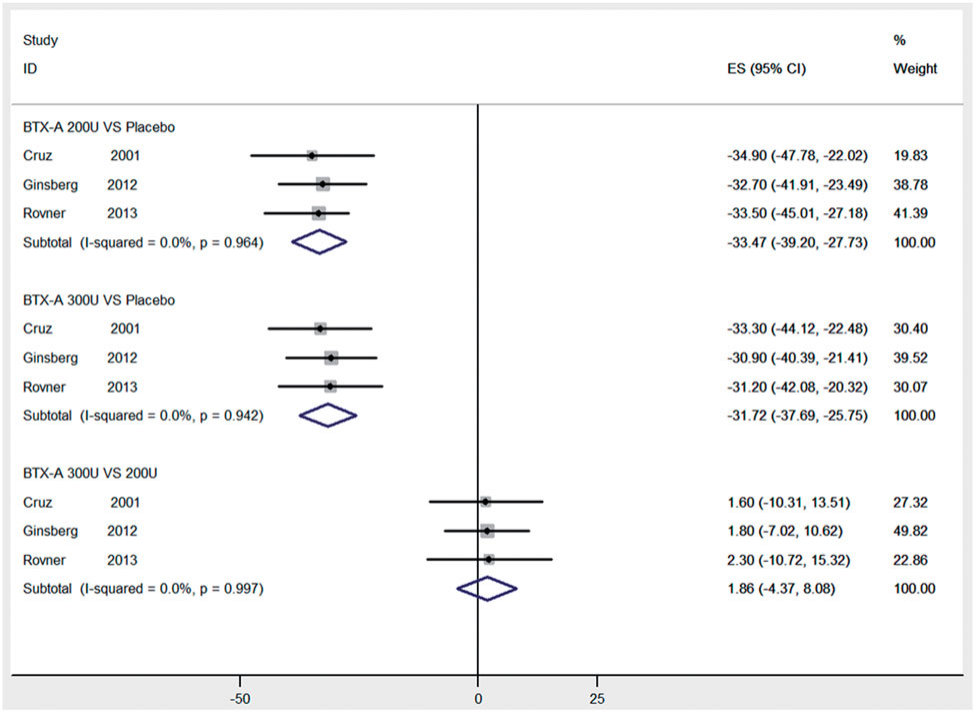
Forest plot of the changes of maximum detrusor pressure at week 6.

Three RCTs [12,14,18] were identified as the MDP. Figure 5 showed that it were statistically significant improvement in MCC at week 6 in a comparison of BTX-A with 300 and 200 U with placebo (MD: −31.72, 95% CI: −37.69 to −25.75; MD: −33.46, 95% CI: −39.74 to −27.18, respectively). Significant improvements were not seen in a comparison of BTX-A with 300 U and BTX-A with 200 U (MD: 1.86, 95% CI: −4.37 to 8.09).

### Safety

**Figure 6.**
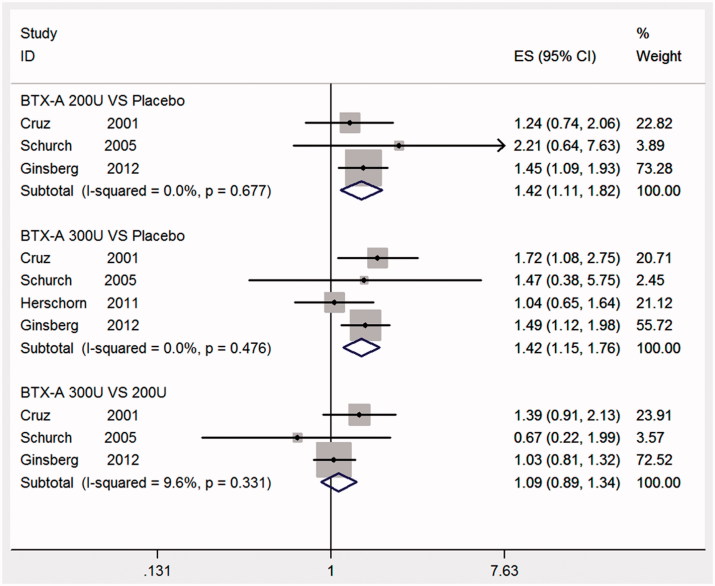
Forest plot of urinary tract infections.

Four studies [11–14] reported that the adverse events were either transient or easily manageable, including the rate of urinary retention, hematuria, muscle weakness, and UTI, which was the most frequently analyzed side effect in this meta-analysis. The subgroups were shown in Figure 6. The results for 300 and 200 U compared to placebo were significant and are clearly apparent in Figure 6 (BTX-A with 300 U vs. placebo, RR: 1.42, 95% CI: 1.15–1.76; BTX-A with 200 U vs. placebo, RR: 1.42, 95% CI: 1.11–1.82, respectively). However, there were no differences between BTX-A 300 and 200 U (RR: 1.09, 95% CI: 0.89–1.34) L.

### Publication bias

**Figure 7.**
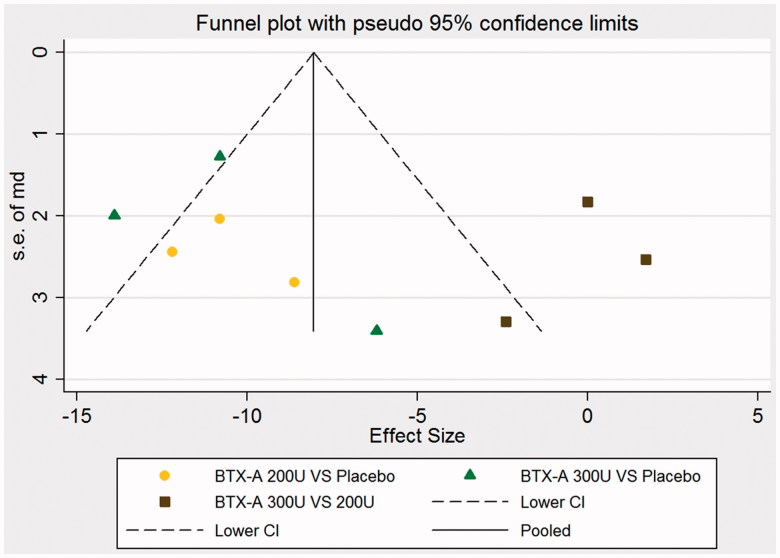
Funnel plot of the changes of urinary incontinence episodes per week at week 6.

No publication bias was observed based on the symmetry of the funnel plot for the frequency of UI per week at week 6, shown in Figure 7. The result of Egger’s test showed there was not a significant difference for UI episodes per week at week 6 (BTX-A with 200 U vs. placebo, Bias = −5.60 [−15.23, 5.42], *p* = 0.322; BTX-A with 300 U vs. placebo, Bias = −1.44 [−5.03, 4.74], *p* = 0.564; BTX-A with 300 U vs. BTX-A with 200 U, Bias = −0.75 [−5.63, 1.44], *p* = 0.662), for MCC at week 6 (BTX-A with 200 U vs. placebo, Bias = 3.42 [1.85, 5.34], *p* = 0.368; BTX-A with 300 U vs. placebo, Bias = 2.35 [−5.42, 4.77], *p* = 0.684; BTX-A with 300 U vs. BTX-A with 200 U, Bias = 0.381 [−3.63, 3.798], *p* = 0.244), for MDP at week 6 (BTX-A with 200 U vs. placebo, Bias = −3.88 [−5.85, 0.34], *p* = 0.097; BTX-A with 300 U vs. placebo, Bias = −5.69 [−15.42, 5.01], *p* = 0.147; BTX-A with 300 U vs. BTX-A with 200 U, Bias = −0.785 [−1.55, 2.55], *p* = 0.581), and for UTI (BTX-A with 200 U vs. placebo, Bias = 6.98 [−1.85, 13.99], *p* = 0.781; BTX-A with 300 U vs. placebo, Bias = 2.88 [−5.42, 9.77], *p* = 0.779; BTX-A with 300 U vs. BTX-A with 200 U, Bias = 5.66 [−2.87, 12.65], *p* = 0.785).

## Discussion

NDO, whose quality of life is greatly reduced [19], can be managed by multiple interventions, including bladder and behavioral training, biofeedback, electrical stimulation, botulinum toxin administration, surgery, or pharmacotherapy [20,21]. The current first-line pharmacotherapeutic treatment options indicated for NDO are anticholinergics. However, NDO patients may show a suboptimal response or find that anti-muscarinic therapy is limited by associated adverse events [22,23]. Hence, there is an urgent need for new treatment modalities for NDO. BTX-A mainly inhibits the release of acetylcholine at nerve terminals and paralyzes the detrusor, thereby improving bladder conditions and reducing urinary symptoms [24]. Onabotulinum toxin A was therefore predicted to be an effective treatment for NDO.

The results of effectiveness and safety from BTX-A, with 200 U and with 300 U, as well as placebo, were investigated. Comparing with the placebo, all outcomes, including frequency of UI per day and per week (except in week 2), MCC, and MDP, were significant for BTX-A with 300 U or with 200 U. These results revealed that BTX-A significantly improved UI caused by NDO. The efficacy of BTX-A on UI may be explained by the theory that BTX-A targets both the afferent and efferent neurons and inhibits the release of acetylcholine and other neurotransmitters at nerve terminals, thereby paralyzing the detrusor. However, no significant differences were found between BTX-A with 300 U and with 200 U on all outcomes analyzed in this meta-analysis, consistent with the results of the meta-analysis by Cheng [25] and Gu [26].

All RCTs included in this meta-analysis reported adverse events, especially UTI. Therefore, the aspect of UTI was analyzed to assess the safety of BTX-A. Our study found that the BTX-A with 300 U and with 200 U had slightly more adverse events than the placebo. However, treatment with BTX-A was well-tolerated, and UTI was mainly limited to local regions of the urinary tract. These results were the same as those found in other meta-analyses by Cheng [25], Zhou [20], and Gu [26].

Included studies of our meta-analysis were all randomized double-blind, placebo-controlled trials. The quality of the individual studies included in this study conformed to the quality assessment which we developed. The results of meta-analysis carry great importance from a scientific standpoint but also in clinical practice. However, several potential limitations should be considered in this meta-analysis. First, only five RCTs with a limited number of patients assessing the efficacy and safety of onabotulinum toxin A were included, and these insufficient data may affect the stability of results and final conclusion. Second, although the pathogenesis of OAB in male and female patient is different, such as benign prostatic hyperplasia, is a prominent disease in male patients, may lead to OAB, the subgroup analyses based on the different cannot be performed because of unable to the individual patient data [16]. Finally, the long-term safety, efficacy, and persistence of BTX-A cannot be extrapolated from this article. More high-quality studies with larger sample sizes are needed for assessment of the efficacy and safety of different doses of BTX-A for the treatment of NDO.

## Conclusion

This meta-analysis suggests that treatment with BTX-A is effective and safe for NDO, and recommends use BTX-A with 300 U or with 200 U, as suitable dosage.
